# Facial trauma education in radiology: using surgeon feedback as the benchmark for success

**DOI:** 10.1007/s10140-024-02288-0

**Published:** 2024-10-16

**Authors:** William T. Malouf, Geeth Kondaveeti, Jacline G. Phillips, Kunjan Patel, Justin A. Hall, Torrey L. Fourrier, Nelson May, Nuwan T. Meegalla, Kevin J. Reger, Christopher M. Runyan, Kevin D. Hiatt

**Affiliations:** 1 Department of Radiology, Wake Forest University School of Medicine, Winston Salem, NC USA; 2 Department of Otolaryngology, Wake Forest University School of Medicine, Winston Salem, NC USA; 3 Department of Otolaryngology, LSU-Health Shreveport, Shreveport, LA USA; 4 Department of Plastic and Reconstructive Surgery, Wake Forest University School of Medicine, Winston Salem, NC USA

**Keywords:** Facial trauma, Multidisciplinary education

## Abstract

**Rationale and objectives:**

Interpreting CT studies of facial trauma is challenging, and there are often substantial differences in the characterization of complex facial trauma between radiologists and surgeons. We designed a collaborative multidisciplinary project to reconcile differences in facial fracture interpretation through an educational intervention. The effectiveness of this intervention was evaluated through surgeon feedback on radiology reports.

**Materials and methods:**

Radiology residents, neuroradiology fellows, and neuroradiology attendings were recruited as participants at a single tertiary care academic center. Otolaryngology residents were recruited as evaluators. Participants completed surveys and provided preliminary reports for example cases of facial trauma before and after attending an educational session. Evaluators performed a blinded review of these preliminary reports based on ideal reports developed by surgical and neuroradiology attendings.

**Results:**

26 participants (20 residents, 1 neuroradiology fellow, 5 neuroradiology attendings) completed the study. Six otolaryngology residents participated as evaluators. To assess interrater reliability, three evaluators graded a shared set of 15 reports and demonstrated substantial agreement with a Kendall’s W of 0.71. Participants demonstrated significant improvement in overall report accuracy, clarity, and organization. In subunit analysis, there were significant improvements in reporting Le Fort, nasoseptal, and nasoorbitoethmoid fractures. No significant improvements occurred in the reporting of upper face, zygomaticomaxillary complex, or mandibular fractures. In contrast, survey analysis demonstrated significantly improved confidence in interpreting trauma involving all facial subunits.

**Conclusion:**

Compared with survey results, surgeon assessment of radiology reports better demonstrated areas of improvement after an educational intervention. A multidisciplinary approach to assessing educational efforts may better evaluate the practical effectiveness of educational interventions.

**Supplementary Information:**

The online version contains supplementary material available at 10.1007/s10140-024-02288-0.

## Introduction

Identification and accurate characterization of facial trauma is imperative to guide timely management of life-threatening and permanently debilitating complications. Accurate initial diagnosis helps plan optimally timed interventions to preserve long-term facial cosmesis and function [1–4]. CT is far superior to the physical exam in characterizing facial fractures, which places the radiologist at a central role in the triage and treatment planning for these patients [2, 3, 5].

The CT assessment of facial trauma has been recognized as an area where radiology reporting is commonly dissimilar with surgical classification and management. In particular, radiology reports often fail to identify and appropriately name surgically relevant fracture groups, such as zygomaticomaxillary complex (ZMC) and nasoorbitoethmoid (NOE) fractures [4, 6, 7]. For example, in a prior study evaluating 356 cases of ZMC fractures, nearly one third of cases had concomitant NOE fractures, none of which were described in the radiology reports [7]. Deficiencies in the accuracy and actionability of radiology reporting pose the risks of suboptimal patient care and the loss of confidence in radiologists’ interpretations by ordering physicians [8, 9]. Additionally, characterizing facial trauma using known fracture complexes often results in faster and clearer dictations since a single fracture complex can describe multiple individual fracture planes. Ideally, radiology reports should be accurate, clinically-relevant, and should communicate findings using language and organization that is familiar to referring clinicians [10]. Directed education drawing from multidisciplinary collaboration may be the key to improving the value of radiology reporting [8, 9, 11].

Many educational strategies exist for teaching complex topics like facial trauma [12–14]; however, to our knowledge, there are no studies directly utilizing referring clinician feedback to measure the effectiveness of radiology facial trauma education. In collaboration with our otolaryngology and plastic surgery colleagues, we devised an educational intervention and gauged its effectiveness using surgeon feedback on participants’ performance interpreting a curated group of assessment cases. We hypothesized that the surgeons’ assessment of improvements would differ from data gained through pre- and post-survey responses from participants. An improved assessment of educational outcomes would establish clinician feedback as an important mechanism for the development and refinement of educational interventions, particularly when compared with participant surveys.

## Methods

### Recruitment

This study protocol was approved by our institutional review board. Participants were voluntarily prospectively enrolled from a radiology residency, neuroradiology fellowship, and neuroradiology faculty at a single tertiary care academic medical center. Enrollment was open to radiology residents at all levels of training. Because the project was conducted in the spring, all participating residents had at least 9 months of residency training, including at least 6 weeks of neurologic CT and 1 week of night float call experience at the start of the project. Each participant agreed to participate in the pre-assessment, pre-survey, educational session, post-assessment, and post-survey. Each participant was assigned a unique numerical identification, and identifying information was kept on a separate spreadsheet so that data analyses could be performed blinded to identifying information.

Evaluators were voluntarily recruited from the same institution’s otolaryngology residency program. To qualify for participation, evaluators had to be in their second postgraduate year of training (PGY-2) or higher and had to have participated in facial trauma call and facial trauma surgery. Evaluators agreed to provide blinded assessments of participants’ reports based on a provided rubric and answer key.

### Development of educational materials

Two neuroradiologists (KDH, WTM) collaborated to create a one-hour educational session on facial trauma, incorporating input from a plastic surgeon with facial trauma expertise (CMR). In keeping with common surgical characterization schemes, complex facial trauma was organized into upper, middle, and lower thirds with the midface comprised of 5 functional subunits (nasoseptal, NOE, ZMC, internal orbit, and occlusion-bearing maxillary). Upper face and midface trauma, including discussion of the midface subunits, were covered by the lecture. The lower face (mandible) was not included in this educational session, in part due to time constraints and also to serve as a control for the pre- and post-participation analyses discussed below.

The educational session was scheduled over the noon hour on two separate dates in a two-week period to maximize the potential for participation. The session consisted of an hour-long lecture utilizing PowerPoint (Microsoft Inc., Redmond, WA, USA) in a format typical for the regular noon hour educational sessions delivered at our institution. There were no competing lectures during these periods. No food or other incentives were provided. Nonparticipants were permitted to attend these sessions.

### Surveys

**Fig. 1:**
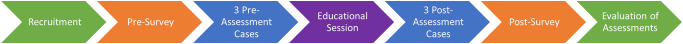
Graphical chronology of the project design

Pre- and post-surveys were designed to assess participants’ perceived competence and comfort with interpreting complex facial trauma on unenhanced CT overall and by individual facial subunits. The individual facial subunits included were: upper face, zygomaticomaxillary complex (ZMC), nasoorbitoethmoid complex (NOE), internal orbit, nasoseptal, and lower face, in keeping with those defined in the literature. Le Fort was also listed as a subunit, as this classification plays a role in defining the occlusion-bearing fragment of midface fractures and is useful in the clear communication of midface trauma [2, 5, 15]. Surveys included a reporting of participants’ familiarity with the referring surgeons’ expectations from radiology reporting of facial trauma and the perceived actionability of their current reporting styles. Participants were instructed to complete the pre-survey prior to starting the pre-assessment cases and to complete the post-survey upon completion of the post-assessment cases (Fig. 1).

### Assessments

**Fig. 2:**
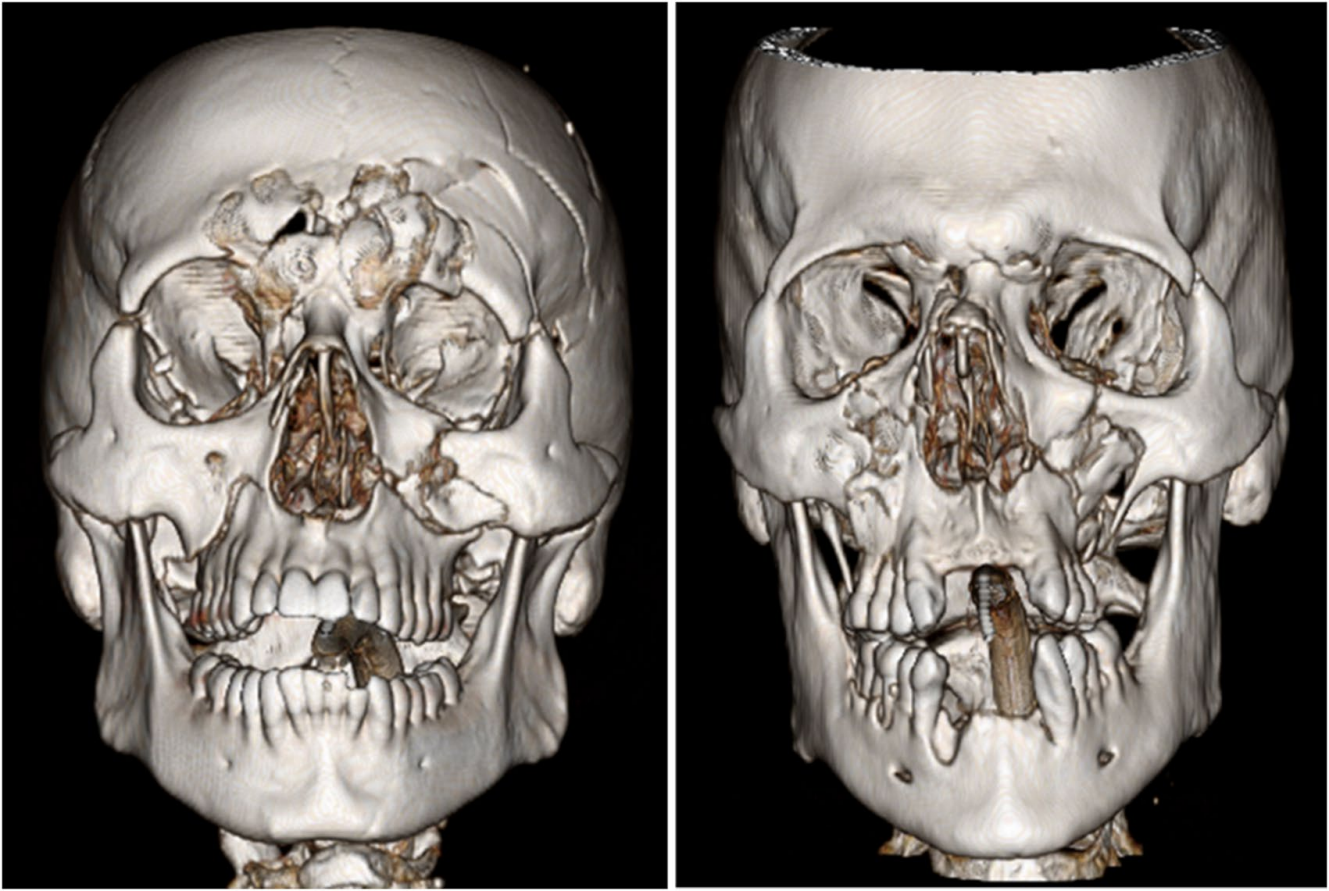
3D rendered CT images from the second pair of assessment cases. Both cases demonstrate LeFort, NOE, and frontal sinus fractures with different degrees of comminution and levels of involvement. These cases were randomized to pre- and post-lecture assessments to evaluate for improved reporting of these subunits

For the pre- and post-assessments, 6 complex facial trauma cases were selected, comprising 3 pairs of similar cases. All cases were unenhanced face CTs including standard algorithm axial images, thin bone algorithm (0.625 mm slice thickness) axial images, and coronal and sagittal bone algorithm reformatted images. The first pair was selected specifically to include both multilevel Le Fort fractures and mandibular fractures, the second to include Le Fort fractures and NOE fractures, and the third to include internal orbital fractures. Representative 3D rendered CT images from the second pair cases are shown in Fig. 2. All cases were deidentified and made available in a shared folder on our institutional PACS (IntelliSpace PACS, Philips, Amsterdam, The Netherlands). Accessing cases in this environment allowed participants to interpret the case in the same manner as their normal daily workflow and enabled them to use the paired 3D rendering software (TeraRecon, Foster City, CA, USA) as needed to aid with interpretation.

Participants were assigned 1 case from each pair (3 total) as pre-assessment cases and the other case from each pair (3 total) as post-assessment cases. They were instructed to complete the pre-assessment cases within a 3-week period preceding the educational session and to complete the post-assessment cases within a 3-week period following the educational session (Fig. 1). Assignments were made such that each case was equally used as a pre- and post-assessment case across participants. For each case, participants were instructed to input their numerical ID, the case number, and a report in free text format into a standard data collection form made using Microsoft Forms (Microsoft Inc., Redmond, WA, USA).

### Report grading

For each of the 6 assessment cases, an answer key was created through the joint efforts of an attending neuroradiologist (KDH) and an attending plastic surgeon who specializes in facial reconstructive surgery (CMR). After collection of all pre- and post-assessment data, evaluators were each provided a set of cases anonymized to participant information and to whether the case was taken as part of a pre- or post-assessment. For each case, they were instructed to use the provided answer key to evaluate the accuracy and clarity of the description of each facial subunit and to assess the overall report accuracy, clarity, actionability, and organization (Table 1). Each metric was rated on a 5-point Likert scale. Evaluators compared the participant reports against the provided answer key and did not review the CT images.

**Table 1:** Grading rubric for assessment cases. Each category was assigned a score of 1-Not at all, 2-Not very, 3-Somewhat, 4-Very, or 5-Perfect

Facial subunit	Upper face	Le Fort	Nasoseptal	NOE	ZMC	Internal orbit	Mandible	Overall
Accurate								
Clear								
Actionable								
Well-organized								

NOE: nasoorbitoethmoid, ZMC: zygomaticomaxillary complex

### Interrater agreement

Evaluators were asked to independently assess an interrater agreement set of 15 reports in addition to their assigned sets. This set included at least 2 reports for each of the 6 unknown cases and included 8 pre-assessment and 7 post-assessment reports submitted by participants distributed across the spectrum of levels of training. Grades assigned for each of these 15 reports were used to assess interrater agreement.

### Data analysis

Pooled group responses on pre- and post-surveys and performance on pre- and post-assessments were compared using the Wilcoxon signed-rank test. Kendall’s W was selected to test interrater reliability. Statistical analyses were conducted using R, version 4.2.1 (R Project for Statistical Computing), with statistical significance defined as *p* < 0.05.

## Results

### Recruitment

Thirty-two participants initially enrolled in the study and 26 completed all study components, consisting of 20 radiology residents (8 first-years, 6 second-years, 4 third-years, and 2 fourth-years), 1 neuroradiology fellow, and 5 neuroradiology attendings. Six of the participants were female (23.1%), which approximately matched the percentage of females among potential participants. Exactly half of the participants attended each educational session offering.

### Surveys

**Fig. 3:**
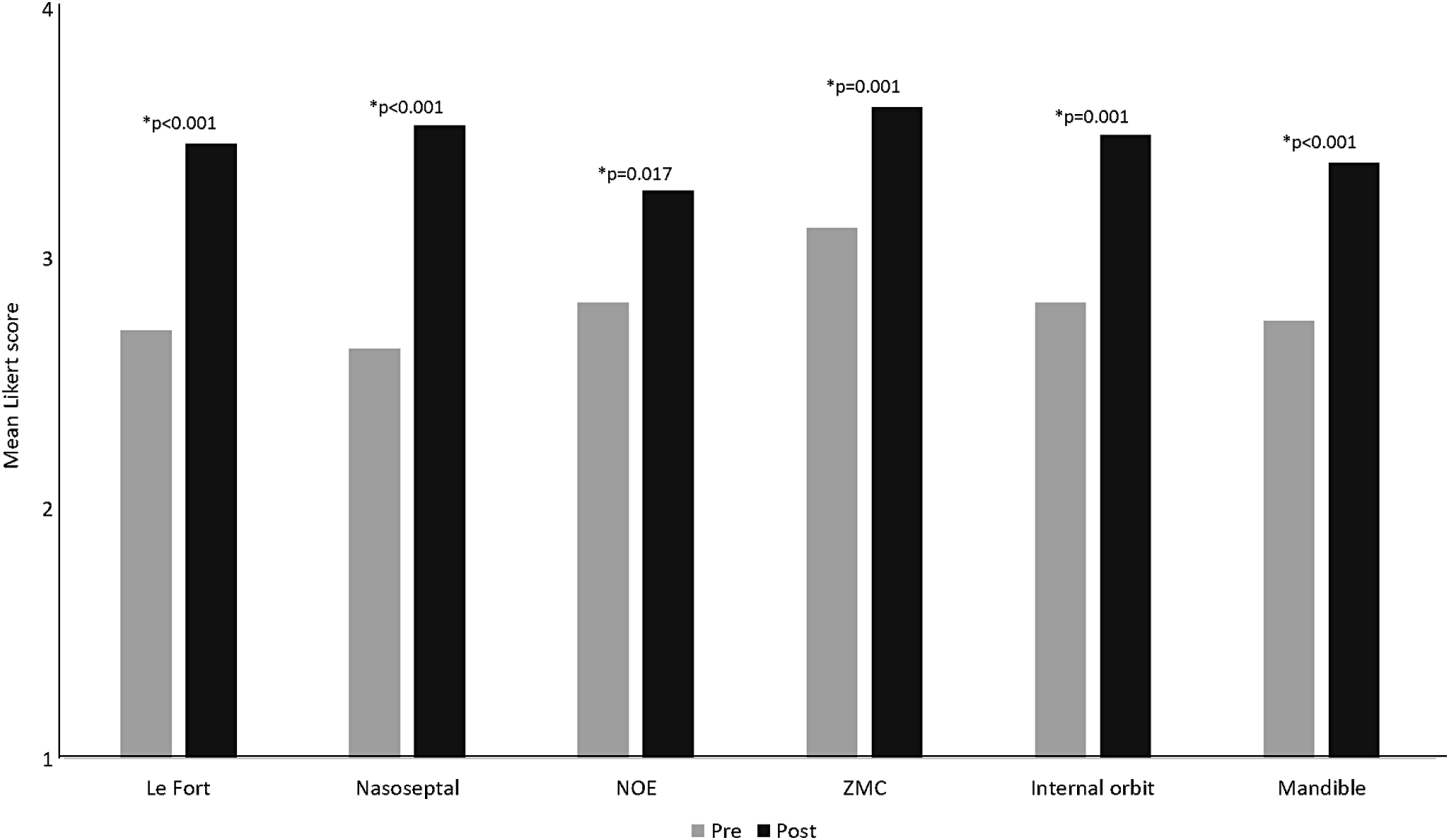
Participant confidence in reporting facial subunits measured by pre- and post-survey. Survey analysis demonstrates significant improvements in the reporting of all facial subunits on a 4-point Likert scale, with asterisks indicating *p* < 0.05 and specific p values as annotated. NOE: nasoorbitoethmoid, ZMC: zygomaticomaxillary complex

Comparing pre- and post-surveys, participants indicated significantly improved confidence with reporting CT studies of complex facial trauma and significantly improved awareness of surgeon’s expectations from imaging reports (Fig. 3). They also reported significantly increased value placed on routinely referring to 3D reformatted images to aid in the evaluation of complex facial trauma cases (*p* = 0.007). The majority of participants, 23 of 26 (88.5%), indicated that NOE fractures were the facial fracture subunit they had been reporting least correctly prior to the educational session.

### Grading and interrater agreement

Six otolaryngology residents participated as evaluators, of which 3 were PGY-2, 1 was PGY-3, 1 was PGY-4, and 1 was PGY-5. Evaluators graded between 15 and 40 reports each, with this unequal distribution necessitated by differences in schedule constraints and clinical duties. Three of the evaluators (2 PGY-2 and 1 PGY-3) additionally completed the interrater agreement set to be used for assessment of interrater agreement. For the 3 evaluators who assessed the same set of 15 reports, a Kendall’s W coefficient of 0.71 was calculated, indicating substantial agreement.

### Assessments

**Fig. 4:**
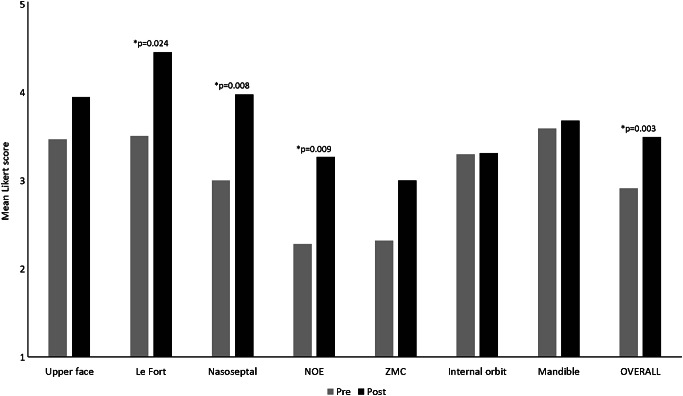
Report accuracy comparisons for all participants between pre- and post-assessments. There were significant improvements in the reporting of Le Fort, nasoseptal, and NOE subunits as well as in the overall report accuracy score on a 5-point Likert scale, with p values < 0.05 marked with an asterisk and specific p values as annotated. NOE: nasoorbitoethmoid, ZMC: zygomaticomaxillary complex

On analysis of assessments graded by the evaluators, the ZMC and NOE subunits had the lowest mean scores on pre-assessments. Comparing pooled post-assessments to pre-assessments, significant improvements were noted in the reporting of the Le Fort, nasoseptal, and NOE subunits as well as in the overall report scores for both report accuracy (Fig. 4) and report clarity. No significant improvements were noted in the assessment of accuracy or clarity of reporting of upper face, ZMC, or internal orbital fractures despite these topics being covered in the educational session. There was also no significant improvement in reporting mandibular fractures; however, this was expected as this topic was not included in the educational intervention to serve as a control for areas of true improvement. The overall report organization metric significantly improved from a mean Likert score of 3.16 on the pre-assessment to 3.48 on the post-assessment (*p* = 0.036). The overall report actionability metric improved, but not significantly from a mean Likert score of 3.16 on the pre-assessment to 3.37 on the post-assessment (*p* = 0.088).

## Discussion

The effective diagnosis and management of complex facial trauma relies on accurate radiology reports that are easy for referring physicians to comprehend and act upon [8, 9]. Unfortunately, significant discrepancies are known to exist in the reporting of facial trauma between radiologists and surgeons, risking debilitating consequences to patients [4, 6, 7]. While radiologists reliably identify individual fractures, they less commonly identify and name fracture patterns important to surgeons. In concordance with previous research [4, 7], the radiology trainees and attendings participating in our study performed poorest in their descriptions of ZMC and NOE fractures before our educational intervention. Furthermore, most participants (88.5%) identified NOE fractures as the subunit they had previously been reporting least correctly.

In our study, the educational outcomes measured by pre- and post-surveys provide an interesting contrast to outcomes measured by performance on assessment cases. When surveyed, participants reported significant improvements in their skill in reporting all facial trauma subunits. Interestingly, this included significantly increased confidence in mandibular trauma, even though this topic was not covered in the educational session due to time constraints as well as to serve as a control for our educational intervention. We suspect that this finding reflects the presence of acquiescence bias, which is a type of response bias in which participants offer favorable responses in support of a research initiative rather than responses matching their true opinions. Differentiating the effects of true, clinically important educational gains from false inflation by acquiescence bias weakens the value of survey-based research; although, acquiescence scores can be computed to help correct for this bias in studies relying on survey data [16].

Our study also measured educational outcomes based on the participants’ performance on assessment cases graded by our surgical colleagues in a blinded fashion. This outcome metric better identified areas of improvement and non-improvement following our educational intervention. When graded by our surgical colleagues, participants demonstrated significant improvement in overall report accuracy, clarity, and organization in post-assessment cases. Additionally, there were significant improvements in the accuracy and clarity of reporting Le Fort, nasoseptal, and NOE fractures. There was a lack of improvement in reporting mandibular fractures, which was not surprising as this topic was not covered in our educational session, and this absence of improvement in interpreting mandibular trauma suggests that this metric was not susceptible to the acquiescence bias suspected to inflate performance on the survey results. Additional topics covered in the session (the upper face and the ZMC and internal orbit midface subunits) did not demonstrate significant improvements in the participants’ performance on post-session example cases. These unexpected shortcomings suggest that the educational intervention was not effective in improving participants’ reporting of these particular subunits. By identifying these specific deficiencies in our educational intervention, we can target specific topics that could be improved upon in future iterations. Interestingly, these deficiencies were not detected by participant surveys.

Innovation in educational practices often take the form of new techniques to deliver content, including flipped classroom, problem-based learning, and creation of enduring audiovisual materials which can be reviewed asynchronously [12–14]. Similar creativity is needed in evaluating the effectiveness of educational sessions, with an emphasis on assessing success through the lens of the referring clinician. When developing educational materials for diagnostic radiology trainees, it is important to consider the desired end result: augmenting the value added by radiology reporting to patient care. To this end, educational initiatives should help learners improve their ability to efficiently and accurately diagnose disease *and* communicate actionable findings. Educational outcome metrics that directly assess participants’ performance on example cases can provide a better assessment of educational outcomes compared with measuring participant confidence by survey. While the formal evaluation of reports used in this study is likely to be impractical in many settings, informal feedback from non-radiologist clinicians can help identify deficiencies and gauge progress [11, 17, 18]. Multidisciplinary collaboration in creating structured report templates is an additional tool for improving report clarity and actionability; although, this approach was not evaluated in this investigation.

This study has several limitations. First, the small number of participants at each level of training prevents statistical comparison within and between groups. However, trends at each level of training paralleled the improvements seen in the pooled group analysis. Second, the cases used for the pre- and post-assessments were small in number and not representative of the full spectrum of facial trauma, which limits the ability to draw conclusions on practical gains in reporting facial trauma among participants. Additionally, there was no formal delayed assessment to test the longevity of the educational gains. Third, although the study setup mimicked a radiology workflow familiar to the participants, the participants knew they were taking part in a simulation and therefore likely did not feel the same pressures for report quality or time efficiency that are inherent to clinical radiology. Fourth, the evaluators were not provided formal training on how to grade the reports, which introduces the potential for inaccuracies and inhomogeneity. To help mitigate this potential bias, an effort was made to evenly distribute pre- and post-assessment cases across evaluators. Additionally, although only 3 of the 6 evaluators participated in evaluating the interrater agreement set of 15 reports, analysis of their reports demonstrated substantial agreement. Finally, the educational intervention and grading rubrics were designed for the general purpose of improving communication between radiologists and facial reconstructive surgeons regardless of institution; however, elements of the intervention may have been affected by preferences within our institution’s surgical practice. It should be noted that the fracture patterns described were in agreement with the literature.

## Conclusion

Surgeon evaluation of facial trauma CT reports before and after a radiology educational session revealed areas of improvement and non-improvement that were not identified by pre- and post-survey data. A multidisciplinary approach to evaluating educational materials in radiology offers advantages over traditional surveys in determining the practical strengths and weaknesses of educational efforts.

## Electronic supplementary material

Below is the link to the electronic supplementary material.

Supplementary Material 1

Supplementary Material 2

Supplementary Material 3

## Data Availability

The data that support the findings of this study are available from the corresponding author, WTM, upon reasonable request.
