# Supplementary material for: Facial trauma education in radiology: using surgeon feedback as the benchmark for success
Source: Emerg Radiol. 2024 Oct 16;31(6):807–14. doi: 10.1007/s10140-024-02288-0 (PMC11625054; doi:10.1007/s10140-024-02288-0)
Supplement: Supplementary file 1 — Supplementary Material 1 [file 10140_2024_2288_MOESM1_ESM.pdf]

# Facial trauma pre-survey

Please complete this survey PRIOR to attending your assigned lecture slot

* Required

1. Enter your participant ID *

2. Have you previously attended a dedicated lecture on facial trauma imaging? *

- ○ Yes – at a conference external to this institution
- ○ Yes – at this institution
- ○ Yes - both at and external to this institution
- ○ No

3. Rate the following: *

| | Very | Somewhat | Not very | Not at all |
|---|---|---|---|---|
| How **confident** do you feel at interpreting facial trauma CTs? | ○ | ○ | ○ | ○ |
| How **aware** do you think you are of what surgeons want to see in a facial trauma CT report? | ○ | ○ | ○ | ○ |
| How **valuable** do you think 3D reformats are in evaluating facial trauma cases? | ○ | ○ | ○ | ○ |

4. Making 3D reformats costs valuable time, especially when on call. How often do you think radiologists should make and refer to 3D reformats for complex facial trauma cases? *

- ◯ Always
- ◯ Usually
- ◯ Only when needed for troubleshooting
- ◯ Only when requested by ENT
- ◯ Never

5. How often do you list specific Le Fort levels (when applicable) in your reports for facial smash cases? *

- ◯ Always
- ◯ Usually
- ◯ Sometimes
- ◯ Rarely
- ◯ Never

6. Rate your confidence with diagnosing and describing each of the following fracture groups: *

| | Very confident | Somewhat confident | Not very confident | Not at all confident |
|---|---|---|---|---|
| Le Fort | ◯ | ◯ | ◯ | ◯ |
| Zygomaticomaxillary complex (ZMC) | ◯ | ◯ | ◯ | ◯ |
| Nasoorbitoethmoid (NOE) | ◯ | ◯ | ◯ | ◯ |
| Internal orbit | ◯ | ◯ | ◯ | ◯ |
| Nasoseptal | ◯ | ◯ | ◯ | ◯ |
| Mandibular | ◯ | ◯ | ◯ | ◯ |

This content is neither created nor endorsed by Microsoft. The data you submit will be sent to the form owner.

Microsoft Forms
