# Supplementary material for: Facial trauma education in radiology: using surgeon feedback as the benchmark for success
Source: Emerg Radiol. 2024 Oct 16;31(6):807–14. doi: 10.1007/s10140-024-02288-0 (PMC11625054; doi:10.1007/s10140-024-02288-0)
Supplement: Supplementary file 2 — Supplementary Material 2 [file 10140_2024_2288_MOESM2_ESM.pdf]

# Facial trauma post-survey

Please complete this survey AFTER attending your assigned lecture slot

* Required

1. Enter your participant ID *

2. Rate the following: *

| | Very | Somewhat | Not very | Not at all |
|---|---|---|---|---|
| How **confident** do you feel at interpreting facial trauma CTs? | ◯ | ◯ | ◯ | ◯ |
| How **aware** do you think you are of what surgeons want to see in a facial trauma CT report? | ◯ | ◯ | ◯ | ◯ |
| How **valuable** do you think 3D reformats are in evaluating facial trauma cases? | ◯ | ◯ | ◯ | ◯ |

3. Making 3D reformats costs valuable time, especially when on call. How often do you think radiologists should make and refer to 3D reformats for complex facial trauma cases? *

- ◯ Always
- ◯ Usually
- ◯ Only when needed for troubleshooting
- ◯ Only when requested by ENT
- ◯ Never

4. Rate your confidence with diagnosing and describing each of the following fracture groups: *

| | Very confident | Somewhat confident | Not very confident | Not at all confident |
|---|---|---|---|---|
| Le Fort | ◯ | ◯ | ◯ | ◯ |
| Zygomaticomaxillary complex (ZMC) | ◯ | ◯ | ◯ | ◯ |
| Nasoorbitoethmoid (NOE) | ◯ | ◯ | ◯ | ◯ |
| Internal orbit | ◯ | ◯ | ◯ | ◯ |
| Nasoseptal | ◯ | ◯ | ◯ | ◯ |
| Mandibular | ◯ | ◯ | ◯ | ◯ |

5. Which of the following fracture groups do you think you were reporting LEAST correctly prior to attending the facial trauma lecture? *

- ◯ Zygomaticomaxillary complex (ZMC)
- ◯ Nasoorbitoethmoid (NOE)
- ◯ Internal orbit
- ◯ Nasoseptal

6. After attending the lecture, how do you expect your report length to change for complex facial trauma cases? *

- ◯ Get longer
- ◯ Stay about the same
- ◯ Get shorter

With regard to the lecture...

7. On a scale from 1 to 9, how much mental effort did the lecture require of you? *

- ◯ 1 (very, very low mental effort)
- ◯ 2
- ◯ 3
- ◯ 4
- ◯ 5
- ◯ 6
- ◯ 7
- ◯ 8
- ◯ 9 (very, very high mental effort)

8. The amount of information on each slide was: *

- ◯ Too much
- ◯ Just right
- ◯ Not enough

9. The number of case examples included was: *

- ◯ Too much
- ◯ Just right
- ◯ Not enough

10. What percent of the material introduced in the lecture do you think you retained? *

- ◯ 90-100%
- ◯ 60-90%
- ◯ 30-60%
- ◯ 0-30%

11. (Optional) How could the lecture have been better?

This content is neither created nor endorsed by Microsoft. The data you submit will be sent to the form owner.

Microsoft Forms
