# Supplementary material for: Facial trauma education in radiology: using surgeon feedback as the benchmark for success
Source: Emerg Radiol. 2024 Oct 16;31(6):807–14. doi: 10.1007/s10140-024-02288-0 (PMC11625054; doi:10.1007/s10140-024-02288-0)
Supplement: Supplementary file 3 — Supplementary Material 3 [file 10140_2024_2288_MOESM3_ESM.docx]

**Sample Answer Key**

**Upper Face**

Mildly comminuted and displaced fractures of the anterior and posterior walls of both frontal sinuses extending into the cribriform plate with small volume pneumocephalus. Soft tissue density fills both frontonasal ducts, but there is no bony occlusion.

**Le Fort**

Bilateral pterygoid plate fractures with midface fractures at the right III and left I and II Le Fort levels. No substantial displacement of the right zygoma.

**Nasoseptal**

Moderately displaced and comminuted fractures of the bilateral nasal bones and maxillary frontal processes. Highly comminuted fractures of the nasal septum with multiple sites of angulation. Mildly displaced fractures of the nasal spine.

**NOE**

Bilateral minimally displaced nasoorbitoethmoid fractures with a single fracture fragment on the right (Markowitz type 1) and mild comminution on the left with the anterior and posterior lacrimal crests remaining on the dominant fracture fragment (Markowitz type 2).

**ZMC**

No ZMC fracture is identified on either side. (Explanation: There is no fracture separating the zygoma from the maxilla on either side.)

**Internal orbit**

On the right, there is displacement of fracture fragments into the superomedial orbital roof and mildly displaced fractures of all internal orbital walls. Small volume extraconal hemorrhage, particularly in the superomedial orbit.

On the left, there are minimally displaced fractures of the medial orbital wall and orbital floor with trace adjacent extraconal hemorrhage.

Symmetric globe contours without proptosis. No herniation or rounding of the extraocular muscles.

**Mandible**

No mandible fracture. The temporomandibular joints are located.

**Other Findings**

Nondisplaced, longitudinally-oriented fracture of the left lateral aspect of the hard palate extending into the left maxillary alveolar ridge involving the distal (lateral) aspect of the left lateral incisor. No dental avulsion.
